# CCN3/NOV Regulates Proliferation and Neuronal Differentiation in Mouse Hippocampal Neural Stem Cells via the Activation of the Notch/PTEN/AKT Pathway

**DOI:** 10.3390/ijms241210324

**Published:** 2023-06-19

**Authors:** Yan Luan, Hanyue Zhang, Kaige Ma, Yingfei Liu, Haixia Lu, Xinlin Chen, Yong Liu, Zhichao Zhang

**Affiliations:** Institute of Neurobiology, Xi’an Jiaotong University Health Science Center, Xi’an 710061, China

**Keywords:** CCN3, hippocampal neural stem cells, proliferation, differentiation, Notch/PTEN/AKT pathway

## Abstract

Neural stem cells (NSCs) persist in the subgranular zone (SGZ) throughout the lifespan and hold immense potential for the repair and regeneration of the central nervous system, including hippocampal-related diseases. Several studies have demonstrated that cellular communication network protein 3 (CCN3) regulates multiple types of stem cells. However, the role of CCN3 in NSCs remains unknown. In this study, we identified CCN3 expression in mouse hippocampal NSCs and observed that supplementing CCN3 improved cell viability in a concentration-dependent manner. Additionally, in vivo results showed that the injection of CCN3 in the dentate gyrus (DG) increased Ki-67- and SOX2-positive cells while decreasing neuron-specific class III beta-tubulin (Tuj1) and doublecortin (DCX)-positive cells. Consistently with the in vivo results, supplementing CCN3 in the medium increased the number of BrdU and Ki-67 cells and the proliferation index but decreased the number of Tuj1 and DCX cells. Conversely, both the in vivo and in vitro knockdown of the *Ccn3* gene in NSCs had opposite effects. Further investigations revealed that CCN3 promoted cleaved Notch1 (NICD) expression, leading to the suppression of PTEN expression and eventual promotion of AKT activation. In contrast, *Ccn3* knockdown inhibited the activation of the Notch/PTEN/AKT pathway. Finally, the effects of changes in CCN3 protein expression on NSC proliferation and differentiation were eliminated by FLI-06 (a Notch inhibitor) and VO-OH (a PTEN inhibitor). Our findings imply that while promoting proliferation, CCN3 inhibits the neuronal differentiation of mouse hippocampal NSCs and that the Notch/PTEN/AKT pathway may be a potential intracellular target of CCN3. Our findings may help develop strategies to enhance the intrinsic potential for brain regeneration after injuries, particularly stem cell treatment for hippocampal-related diseases.

## 1. Introduction

The hippocampus plays a critical role in governing human physiological and behavioral functions, and structural or functional abnormalities within it have been linked to neurological and psychiatric disorders, including epilepsy, Alzheimer’s, schizophrenia, and depression [1,2,3]. The subgranular zone (SGZ) of the dentate gyrus (DG) within the hippocampus contains neural stem cells (NSCs) throughout life, which can proliferate and differentiate into mature neurons, integrate into existing neural networks, and perform biological functions [4]. Recent studies have indicated that NSCs have potential curative effects for hippocampal dysfunction diseases, such as Alzheimer’s disease, depression, and epilepsy, among others [5,6,7]. However, the major setback is the insufficient availability and the directed differentiation of NSCs for stem cell therapy. Thus, it is essential to study how to promote the proliferation and neuronal differentiation of NSCs.

Cellular communication network (CCN) proteins form a family of six cysteine-rich regulatory proteins that function as scaffolding proteins to regulate and maintain the interconnection and balance between diverse signaling pathways [8]. Among these CCN proteins, CCN3, also known as nephroblastoma overexpressed (NOV), is involved in regulating cell proliferation, migration, differentiation, and survival in numerous types of stem cells [9,10,11]. In mesenchymal stem cells (MSCs), CCN3 significantly suppresses osteogenic differentiation by inhibiting the Notch and BMP signaling pathways [12]. Brief CCN3 treatment can increase hematopoietic stem cell (HSC) numbers substantially [13]. However, CCN3 can reduce the SHH-induced proliferation of primary cultured granule neuron precursors (GNPs) [14]. Hence, the function of CCN3 in stem cells remains unclear, and it is necessary to further investigate whether CCN3 plays a role in regulating NSC proliferation and differentiation.

In this study, we investigated the function of CCN3 in mouse hippocampal NSCs and elucidated its potential intracellular mechanism. First, we examined the expression of CCN3 in primary NSCs isolated from neonatal mouse hippocampus. Then, we assessed the effect of CCN3 modulation on the apoptosis, proliferation, and neuronal differentiation of SGZ-derived NSCs both in animals and in NSC culture. Finally, we investigated the involvement of intracellular Notch/PTEN/AKT signaling. Our findings propose a new approach for exploring stem cell treatment strategies for Alzheimer’s disease, depression, epilepsy, and related disorders.

## 2. Results

### 2.1. CCN3 Promotes the Cell Viability of Cultured Neonatal Mouse Hippocampal NSCs

**Figure 1:**
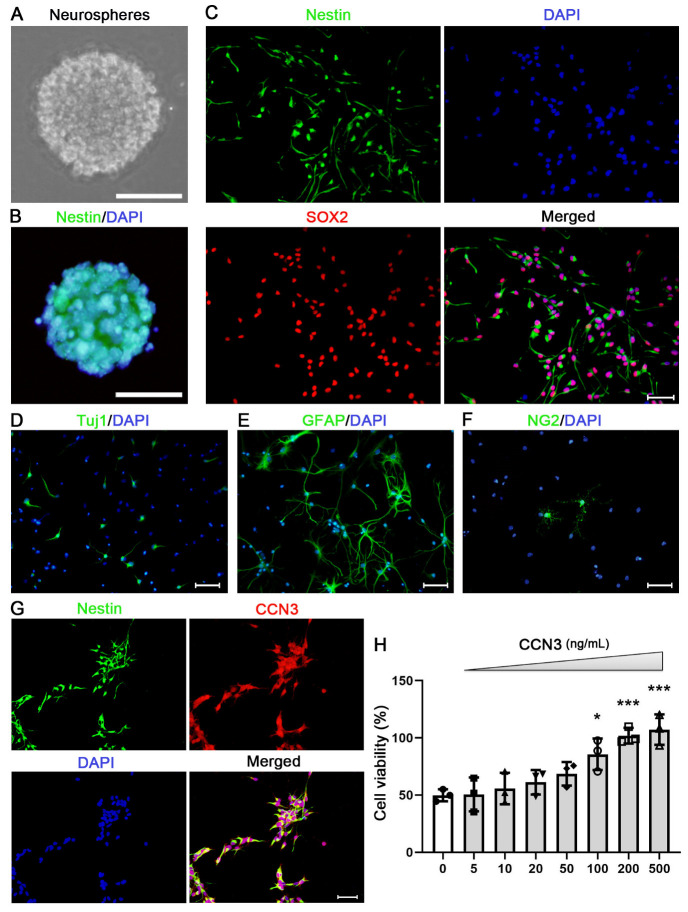
**Culture of mouse hippocampal NSCs and the effect of CCN3 on cell viability.** Neural stem cells (NSCs) were isolated from the hippocampus of neonatal mice. Following 3–5 days of culturing, 90–170 μm neurospheres were observed (**A**) and the majority of these cells expressed nestin (**B**). (**C**) The NSCs were identified using double-immunofluorescent labeling with nestin and SOX2. DAPI was used as a counterstain for the nuclei. (**D**–**F**) Tuj1-, GFAP-, or NG2-positive cells were observed following normal differentiation medium culturing for 3 days. (**G**) CCN3 expression co-localized with nestin, as observed by double immunofluorescent staining. (**H**) NSCs were exposed to varying concentrations of CCN3 (5, 10, 20, 50, 100, 200, and 500 ng/mL) for 3 days, and cell viability was measured using the CCK-8 assay. All results are expressed as the mean ± SD with single data points from at least three independent experiments. Statistical analysis was performed using one-way ANOVA. The Kolmogorov–Smirnov test was used for normality and homogeneity. * *p* < 0.05; *** *p* < 0.001 versus normal control (0 ng/mL). Scale bars in (**A**,**B**) denote 100 μm; in (**C**–**G**), 50 μm.

Primary NSCs were isolated from the neonatal mouse hippocampus. After culturing for 3–5 days, neurospheres (90–170 μm) comprising numerous nestin-positive cells were observed (Figure 1A,B). In vitro culturing yielded a high proportion of nestin-positive cells, with 93.22% ± 4.05% displaying this marker. Furthermore, 98.06% ± 7.08% of these nestin-positive cells were found to express SOX2, as confirmed by double immunofluorescent staining (Figure 1C). To assess the differentiating potential of these hippocampal NSCs, cells were incubated in the differentiation medium (DMEM/F12 supplemented with 1% N2, 2% B27, and 1% FBS). Following three days of culturing, the presence of Tuj1- (a marker for immature neurons), GFAP- (an astrocyte marker), or NG2-positive (an oligodendrocyte progenitor cell marker) cells were observed (Figure 1D–F). The above findings indicated that these cultured cells were primary hippocampal NSCs. Subsequently, double immunofluorescent staining was employed to examine the presence of CCN3 in mouse hippocampal NSCs. As expected, CCN3 co-localized with the NSC marker nestin (Figure 1G). To investigate the role of CCN3 in regulating cell viability, NSCs were exposed to varying concentrations of CCN3 (5, 10, 20, 50, 100, 200, and 500 ng/mL) for 3 days. The increasing CCN3 concentration corresponded to enhanced cell viability (Figure 1H). Given the insignificant difference in the effect of CCN3 at 200 and 500 ng/mL, a specific concentration of 200 ng/mL was chosen for further experimentation.

### 2.2. CCN3 Promotes Nsc Proliferation in Mouse Hippocampus

**Figure 2:**
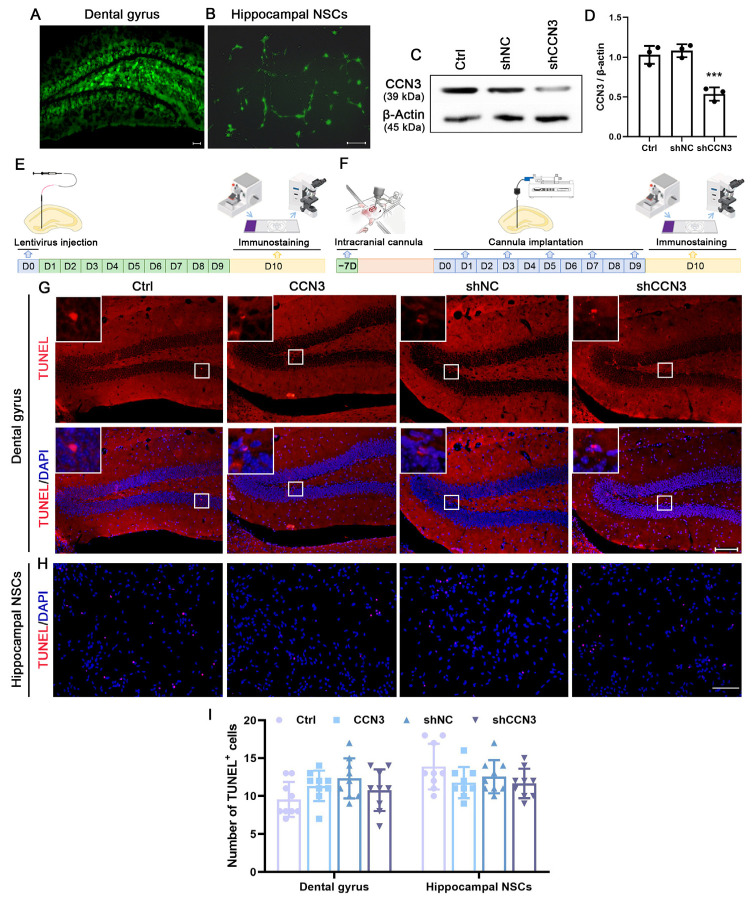
**Alteration of CCN3 expression has little impact on cell apoptosis.** Cells were transfected with either a *Ccn3*-targeting shRNA (shCCN3) or a non-specific shRNA (shNC). After 7 days, eGFP-positive cells were found in both the DG area (**A**) and cultured hippocampal NSCs (**B**). (**C**,**D**) WB band quantification for the ratio of CCN3 to β-Actin was presented, and each value represents the mean ± SD of three independent experiments (*n* = 3). Differences between groups were analyzed using one-way ANOVA, followed by Tukey’s post hoc test. *** *p* < 0.001 versus the shNC group. The schematic workflow of the experiment involving the injection of lentivirus (**E**) and CCN3 protein solution (**F**) is as follows. Mice were randomly assigned to different treatment groups and received a DG infusion of normal saline solution (Ctrl), CCN3 protein solution (CCN3), lentiviral shRNA against *Ccn3* (shCCN3), or control shRNA (shNC). (**G**–**I**) TUNEL staining was utilized to detect apoptotic cells. Data are presented as the percentage of TUNEL-positive cells in the total DAPI-stained cells, and each value represents the mean ± SD of nine independent experiments (*n* = 9). Differences between groups were analyzed using one-way ANOVA, followed by Tukey’s post hoc test. Scale bars in (**A**,**B**,**G**) represent 100 μm; in (**H**), 50 μm.

To investigate the impact of CCN3 on NSC proliferation in the SGZ, we utilized a lentiviral vector to transfect the cells with *Ccn3*-targeted shRNA. After seven days of transfection, eGFP-positive cells were observed in both the DG area and the culture of hippocampal NSCs (Figure 2A,B), subsequently demonstrating a significant reduction in CCN3 expression upon transfection with *Ccn3*-targeting shRNA (Figure 2C,D). Since CCN3 can influence cell viability, we first examined its effect on NSC apoptosis using TUNEL staining. The suppression of CCN3 in NSCs within the DG was achieved via transfection with *Ccn3*-targeting shRNA (Figure 2E). Conversely, an increase in the extracellular concentration of CCN3 was accomplished by injecting a solution of CCN3 protein (Figure 2F). For the cultured primary hippocampal NSCs, lentivirus or a CCN3 protein solution was added to the medium. TUNEL staining revealed no significant variation in apoptotic cells among the different treatments both in vivo and in vitro (Figure 2G–I). These findings indicate that CCN3 does not affect apoptosis in mouse hippocampal NSCs.

**Figure 3:**
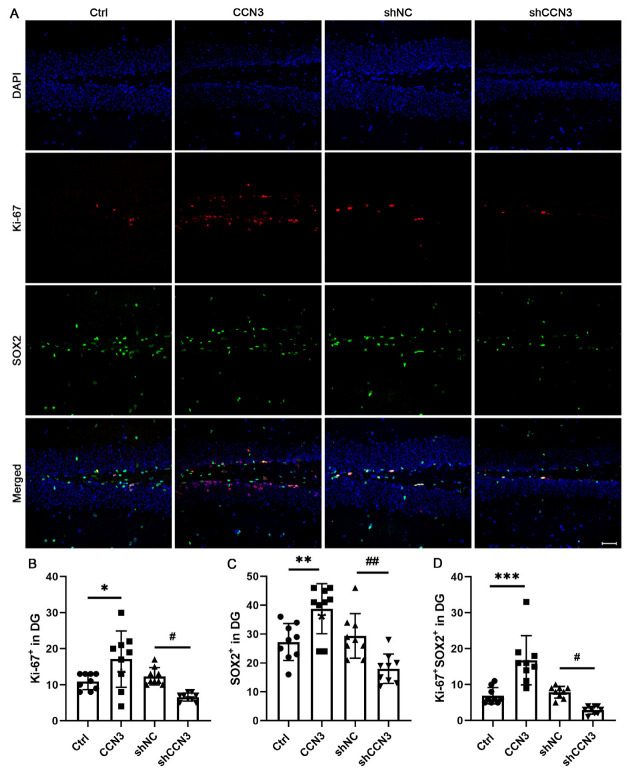
**CCN3 promotes NSC proliferation in the mouse dental gyrus.** (**A**) Following the same grouping as before, Ki-67 and SOX2 double immunostaining was used to detect NSC proliferation. Quantitative analysis of the number of Ki-67 (**B**), SOX2 (**C**), and double-positive cells (**D**), each data represents the mean ± SD from 9 animals (*n* = 9). Scale bar = 50 μm. Differences between groups were analyzed using one-way ANOVA, followed by Tukey’s post hoc test. The Kolmogorov–Smirnov test was used for normality and homogeneity. * *p* < 0.05, ** *p* < 0.05, and *** *p* < 0.001 versus the Ctrl group; ^#^ *p* < 0.05 and ^##^ *p* < 0.001 versus the shNC group.

To assess the impact of CCN3 on NSC proliferation, SOX2 and Ki-67 double immunostaining was performed (Figure 3A). The results revealed no statistical difference between the control and shNC groups. However, it is noteworthy that CCN3 administration led to an increase in both the number of SOX2-positive cells and Ki-67-positive cells. In contrast, inhibiting CCN3 expression significantly reduced the population of SOX2-positive cells and Ki-67-positive cells (Figure 3B–D). These findings strongly suggested that the expression level of CCN3 was related to the proliferation of mouse hippocampal NSCs.

### 2.3. CCN3 Inhibits Neuronal Differentiation of Nscs in Mouse Hippocampus

**Figure 4:**
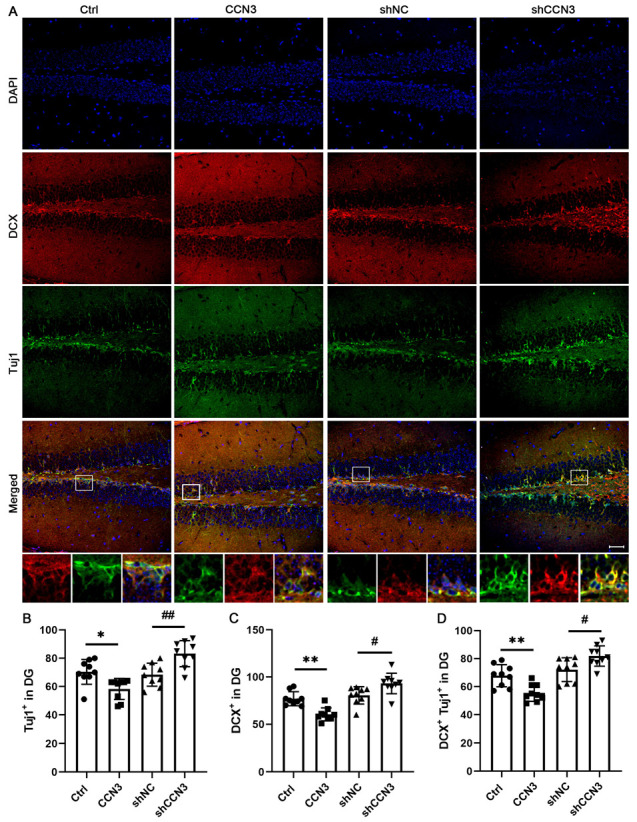
**CCN3 inhibits neuronal differentiation of NSCs in the mouse dental gyrus.** (**A**) Following the same grouping as before, immunostaining was used to detect Tuj1- and DCX- cells. The square frames are enlarged to identify DCX- (red) and Tuj1 (green)-positive cells. (**B**–**D**) All results are expressed as the mean ± SD with single data points from 9 animals (*n* = 9). Scale bar = 50 μm. Differences between groups were analyzed using one-way ANOVA, followed by Tukey’s post hoc test. The Kolmogorov–Smirnov test was used for normality and homogeneity. * *p* < 0.05 and ** *p* < 0.01 versus the Ctrl group; ^#^ *p* < 0.05 and ^##^ *p* < 0.01 versus the shNC group.

To investigate the effect of CCN3 on the neuronal differentiation of hippocampal NSCs, we used neuron-specific class III beta-tubulin (Tuj1) and doublecortin (DCX) as markers to identify immature neurons. Immunofluorescence double-labeling exhibited the co-localization of DCX+ cells with Tuj1 (Figure 4A). As expected, there was no statistical difference between the control and shNC groups. Compared to the control group, exposure to CCN3 significantly decreased the number of Tuj1-positive (from 70.4 ± 8.09 to 58.2 ± 6.92, *p* = 0.0222) and DCX-positive (from 76.9 ± 7.02 to 60.3 ± 6.54, *p* = 0.0018) cells. In contrast, silencing CCN3 significantly increased the number of Tuj1-positive (from 70.4 ± 8.09 to 58.2 ± 6.92, *p* = 0.0222) and DCX-positive (from 76.9 ± 7.02 to 60.3 ± 6.54, *p* = 0.0018) cells compared to the shNC group (Figure 4B–D). These results suggest that CCN3 can inhibit the neuronal differentiation of NSCs in the adult mouse hippocampus.

### 2.4. CCN3 Regulates the Proliferation and Neuronal Differentiation of Cultured Mouse Hippocampal NSCs

**Figure 5:**
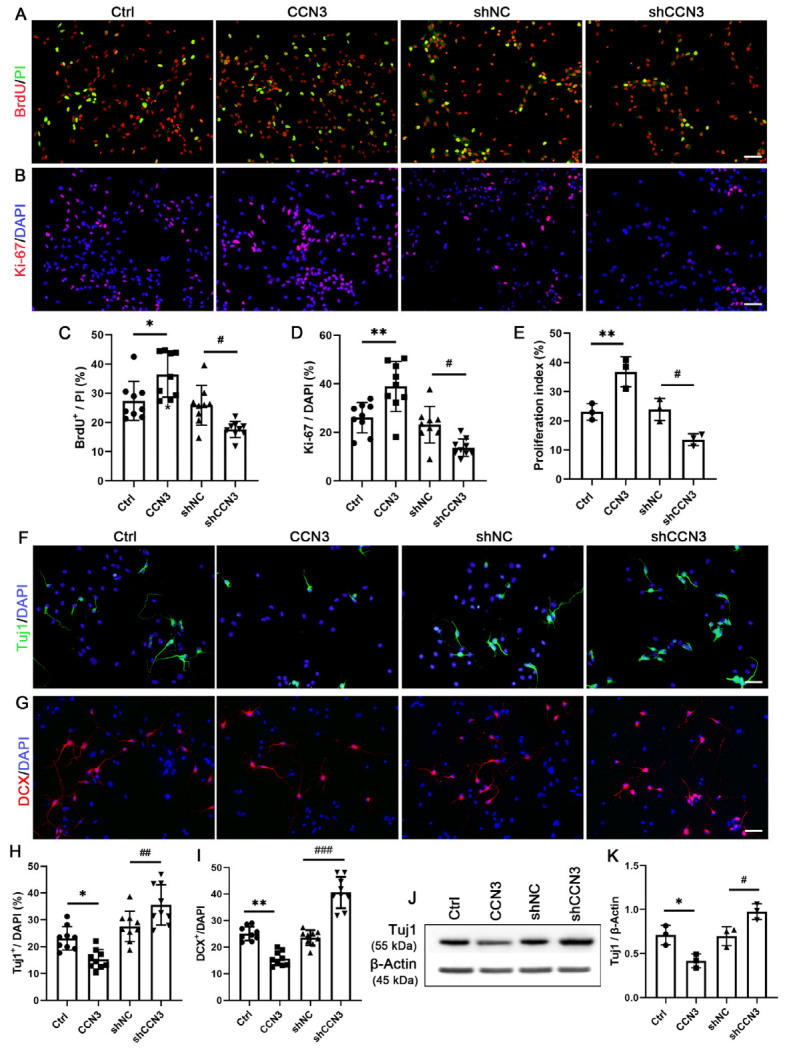
**CCN3 promotes proliferation and inhibits the neuronal differentiation of culture mouse hippocampal NSCs.** The effect of CCN3 on NSC proliferation was measured by BrdU (**A**) and Ki-67 (**B**). Each single data points from nine independent cell culture preparations (*n* = 9) are presented as the percentage of BrdU+ cells among PI-stained cells (**C**) or Ki-67+ cells (**D**) among DAPI-stained cells. (**E**) Cell cycle analysis was used to detect the proliferation index (PI). The result is expressed as the mean ± SD with single data points from three independent cell culture preparations (*n* = 3). The number of Tuj1- (**F**) and DCX- (**G**) positive cells was determined by immunostaining. Each single data points from nine independent cell culture preparations (*n* = 9) are presented as the percentage of Tuj1^+^ (**H**) and DCX^+^ cells (**I**) among DAPI-stained cells. (**J**,**K**) Representative WB images illustrated the protein expression of Tuj1, and β-Actin was used as a reference protein. The ratio of Tuj1 to β-Actin was quantified using WB band analysis, and the result is expressed as the mean ± SD with single data points from three independent cell culture preparations (*n* = 3). Scale bars in (**A**,**B**,**F**,**G**) denote 50 μm. Differences between groups were analyzed using one-way ANOVA, followed by Tukey’s post hoc test. The Kolmogorov–Smirnov test was used for normality and homogeneity. * *p* < 0.05 and ** *p* < 0.01 versus the Ctrl group; ^#^ *p* < 0.05, ^##^ *p* < 0.01, and ^###^ *p* < 0.001 versus the shNC group.

We further investigated the effects of CCN3 on cell proliferation and neuronal differentiation in cultured neonatal mouse hippocampal NSCs. The obtained results were consistent with our previous in vivo observations. Firstly, there was no statistically significant difference between the control and shNC groups. Treatment with CCN3 significantly increased the number of BrdU- and Ki-67-positive cells, while silencing CCN3 exhibited the opposite effect (Figure 5A–D). Additionally, cell cycle analysis demonstrated that CCN3 enhanced the proliferation index (PI), whereas *Ccn3* knockdown reduced the PI (Figure 5E). Furthermore, immunostaining was employed to determine the number of Tuj1- and DCX-positive cells, while Western blotting was used to evaluate the expression levels of Tuj1. The results showed that CCN3 reduced the number of Tuj1- and DCX-positive cells and suppressed Tuj1 expression. In contrast, the knockdown of Ccn3 showed opposite effects with respect to neuronal differentiation (Figure 5F–K). Hence, these results indicate that CCN3 promotes cell proliferation and impedes neuronal differentiation in mouse hippocampal NSCs.

### 2.5. CCN3 Regulates Proliferation and Neuronal Differentiation via Notch/Pten/Akt Pathway

**Figure 6:**
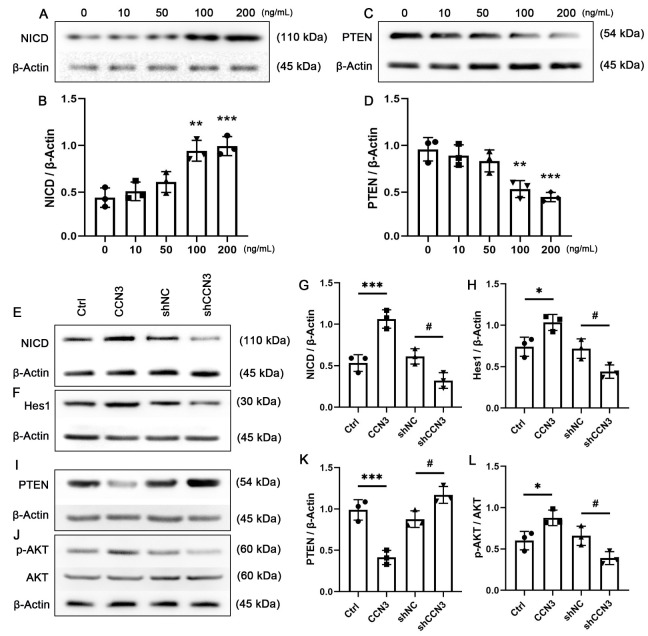
**CCN3 regulates the activation of the Notch/PTEN/AKT axis.** Mouse hippocampal neural stem cells (NSCs) were treated with CCN3 at varying concentrations (10, 50, 100, and 200 ng/mL) for 24 h. (**A**,**C**) A Western blot assay was performed to detect the expression levels of cleaved Notch1 (NICD) and PTEN. (**B**,**D**) The value represents the mean ± SD with single data points from three independent cell culture preparations (*n* = 3). Statistical analysis was performed using one-way ANOVA. The Kolmogorov–Smirnov test was used for normality and homogeneity. ** *p* < 0.01 and *** *p* < 0.001 versus the 0 ng/mL group. The relative ratios of NICD (**E**), Hes1 (**F**), and PTEN (**I**) to β-Actin and p-AKT/AKT (**J**) were quantified by measuring Western blot bands after the modulation of CCN3 expression. (**G**,**H**,**K**,**L**) The value represents the mean ± SD with single data points from three independent cell culture preparations (*n* = 3). Differences between groups were analyzed using one-way ANOVA, followed by Tukey’s post hoc test. The Kolmogorov–Smirnov test was used for normality and homogeneity. * *p* < 0.05 and *** *p* < 0.001 versus the Ctrl group; ^#^ *p* < 0.05 versus the shNC group.

Prior studies have demonstrated that CCN3 plays an important regulatory function in diverse types of cells by activating both Notch and AKT signaling pathways [15,16,17]. To gain a deeper understanding of the role of CCN3 in hippocampal NSCs, we initially evaluated the expression of cleaved Notch1 (NICD, the intracellular domain of Notch protein) and PTEN, a crucial AKT pathway regulator. Cells were manipulated with a series of CCN3 concentrations (10, 50, 100, and 200 ng/mL) for 24 h, and Western blot analysis was performed. The results showed a dose-dependent increase in NICD expression following CCN3 stimulation (Figure 6A,B), whereas PTEN expression decreased under the CCN3 treatment (Figure 6C,D). Additionally, with Western blot analysis, we examined the expression of Hes1, a downstream component of the Notch signaling pathway, and AKT phosphorylation levels after replenishing or knocking down *Ccn3* in cultured NSCs. As expected, the shNC treatment had minimal impact on CCN3 expression. More importantly, CCN3 administration increased the expression of NICD and Hes1 and activated AKT while inhibiting PTEN expression. Conversely, the knockdown of *Ccn3* led to the opposite trend (Figure 6E–L). These findings indicate that CCN3 plays a crucial role in regulating Notch/PTEN/AKT signaling pathways in hippocampal NSCs.

**Figure 7:**
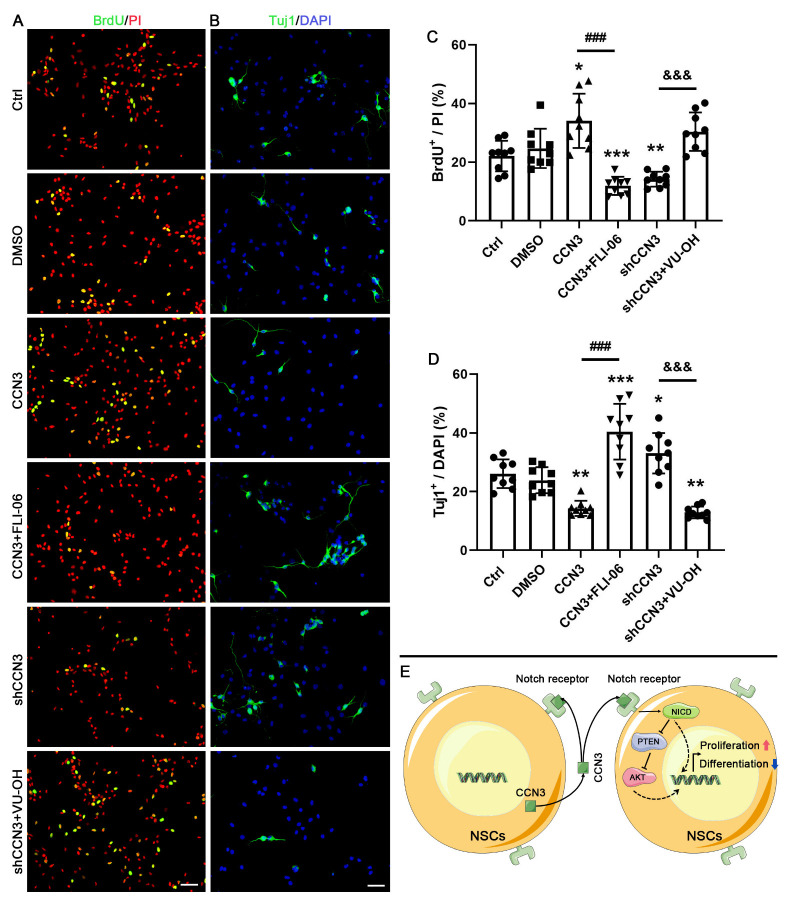
**Notch/PTEN/AKT pathway is closely involved in regulating the effect of CCN3 in mouse hippocampal NSCs.** After the modulation of CCN3 expression, NSCs were treated with a vehicle (DMSO), 20 μM of FLI-06, or 5 μM of VO-OHpic trihydrate (VO-OH). (**A**,**B**) BrdU staining and Tuj1 staining were performed to detect proliferation and neuronal differentiation, respectively. Scale bar = 50 μm. Each single data points from nine independent cell culture preparations (*n* = 9) are presented as the percentage of BrdU^+^ cells among PI-stained cells (**C**) or Tuj1^+^ cells (**D**) among DAPI-stained cells. Differences between groups were analyzed using one-way ANOVA, followed by Tukey’s post hoc test. The Kolmogorov–Smirnov test was used for normality and homogeneity. * *p* < 0.05, ** *p* < 0.01, and *** *p* < 0.01 versus the DMSO group; ^###^ *p* < 0.001 versus the CCN3 group; ^&&&^ *p* < 0.001 versus the shCCN3 group. (**E**) The illustration presents the mechanisms by which CCN3 regulates proliferation and differentiation in NSCs.

To examine whether CCN3 affects NSC proliferation and differentiation via the regulation of the Notch/PTEN/AKT pathway, we administered 20 μM of FLI-06 to inhibit Notch and 5 μM of VO-OHpic trihydrate (VO-OH) to inhibit PTEN. BrdU staining and Tuj1 staining were utilized to detect proliferation and neuronal differentiation, respectively. The results showed that treatment with either FLI-06 or VO-OH inhibited the effects of CCN3 or shCCN3 on hippocampal NSC proliferation and neuronal differentiation (Figure 7A–D). This outcome suggests that the regulation of the activation of the Notch PTEN/AKT signaling pathway may be involved in the modulation of CCN3 on the proliferation and differentiation of hippocampal NSCs.

## 3. Discussion

The process of hippocampal neurogenesis encompasses NSC proliferation, the differentiation of these cells into mature neurons, and their integration into existing neuronal circuits. It is strongly associated with particular forms of learning and memory [18]. More importantly, previous studies have established that impaired neurogenesis contributes to a range of neurological and psychiatric disorders, including depression, Alzheimer’s disease, and epilepsy [19,20]. This highlights the potential for the proper management of hippocampal neurogenesis in treating hippocampal dysfunction. The present study demonstrates the fundamental role of CCN3 in hippocampal neurogenesis. In particular, our findings indicated that CCN3 promotes NSC proliferation while inhibiting neuronal differentiation. Accordingly, CCN3 could represent a distinct intervention point in the regulation of hippocampal neurogenesis. We believe that these findings could have direct implications for the development of future CNS therapeutics. It is important to note that various types of cells, including neurons and astrocytes, have the potential to secrete the CCN3 protein [21,22], which can ultimately affect the proliferation and differentiation of NSCs. Additional research is necessary to validate whether the secretion of CCN3 proteins by these cells actually influences NSCs.

As a member of the CCN family of extracellular matrix-associated proteins, CCN3 plays a role in regulating cell proliferation in various cell types. However, it fulfills different roles in different cell types [23,24]. For example, in breast cancer cells, CCN3 increased cell proliferation by upregulating the expression of cyclin D1 [25]. Similarly, in pancreatic cancer cells, the overexpression of CCN3 facilitated cell proliferation and migration by inducing an epithelial–mesenchymal transition [26]. In contrast, CCN3 can suppress enzalutamide-resistant prostate cancer cell proliferation by inhibiting androgen receptor signaling in prostate cancer cells [27]. In addition, CCN3 has been shown to inhibit the proliferation of glomerular cells by inhibiting PDGF [28]. The possible reason is that CCN3 can interact with various cell surface receptors, including integrins, heparan sulfate proteoglycans, low-density-lipoprotein receptor-related protein 1 (LRP1), and Notch receptors [29,30]. Depending on the target cell type and the biological context, CCN3 fulfills different roles in regulating cell behavior by binding to different receptors. For instance, CCN3 promotes the proliferation of muscle skeletal cells via interactions with integrin β1 [31]. In vascular smooth muscle cells, CCN3 suppresses neointimal thickening by inhibiting cell migration with the activation of Notch1 and Notch3 receptors [32]. Our present study showed that CCN3 remarkably increased the expression of NICD (cleaved Notch1) and Hes1. These results suggested that CCN3 regulated the proliferation and differentiation of mouse hippocampal NSCs via interactions with the Notch1 receptor. However, whether other types of CCN3 receptors are expressed in NSCs and whether these receptors are involved in regulating NSCs need to be investigated in the future.

CCN3 in the cell is not constant, but different roles are played in the regulation of proliferation and differentiation in different cell types via different signaling pathways. Despite previous studies demonstrating that CCN3 could promote cell proliferation and activate NOTCH and AKT pathways [33], little is known about the effect of CCN3 on PTEN expression. In this study, we reported that CCN3 could inhibit PTEN expression in hippocampal NSCs. Upon Notch activation, the expression of Hes1 (a downstream target gene of NICD) is induced. Previous studies indicated that Hes1 could bind to the PTEN promoter region and repress its transcription, leading to a decrease in PTEN expression [34]. As a lipid phosphatase, PTEN specifically increases the phosphatidylinositol (4,5)-bisphosphate (PIP_2_) level by reducing the intracellular level of phosphatidylinositol-3,4,5-trisphosphate (PIP3), which in turn inhibits the AKT signaling pathway [35]. In this study, our findings demonstrate a considerable increase in the expression of NICD and Hes1 expression upon CCN3 supplementation. Conversely, silencing CCN3 had the opposite effect as it resulted in the suppression of NICD and Hes1 expression and the promotion of PTEN expression. More importantly, both FLI-06 (a Notch inhibitor) and VO-OH (a PTEN inhibitor) eliminated the effects of changes in CCN3 protein expression on NSC proliferation and neuronal differentiation. However, we are also aware of some limitations in this study that need to be noted. The changes in HES1 and pAKT were not detected in the presence of FLI-06 and VO-OH. This limitation arises from the fact that various other intracellular signaling molecules can also impact the expression of HES1 and pAKT. From the synthesis of our existing findings, it is reasonable to speculate that CCN3 may play a critical role in the regulation of NSC proliferation and differentiation via the Notch/PTEN/AKT signaling pathway. Specifically, NSCs secrete CCN3 into the stem cell niche, where it binds to a group of receptors, primarily the Notch1 receptor, via paracrine or autocrine pathways. This binding results in the elevation of NICD expression, leading to the suppression of PTEN expression and ultimately promoting AKT activation, which results in cell proliferation while inhibiting neuronal differentiation in the NSCs (Figure 7E). Because CCN3 influences cell behaviors by binding to cell membrane receptors, we did not perform a rescue experiment that involves the inhibition of CCN3 expression on NCSs followed by treatment with CCN3. Nonetheless, we cannot exclude the possibility that CCN3 may interact with intracellular proteins to affect cell behaviors. The mechanism of action of CCN3 requires further research.

In this study, our findings suggested that CCN3 regulated the activation of the Notch/PTEN/AKT signaling pathway, thereby influencing the proliferation and neuronal differentiation of NSCs. The Notch/PTEN/AKT signaling pathway is closely related to the proliferation and differentiation of NSCs [36,37,38]. However, the effects of CCN3 cannot be solely explained by the promotion of Notch/PTEN/AKT signaling pathway activation. Instead, AKT’s biological effects are the result of cross-talks with other signaling pathways, including MAPK [39], Wnt [40], and Shh [41]. For instance, increased phosphorylation levels of AKT lead to the suppression of RAF1 expression and the inhibition of MAPK signaling pathway activation [42]. Phospholipase D1 (PLD1) promotes the Wnt/β-catenin signaling pathway by selectively downregulating ICAT via AKT signaling pathways [43]. Cross-talks among these signaling pathways orchestrate an interconnected biochemical network that regulates cell behaviors and amplifies signaling cascades, ultimately influencing various biological processes. Further studies are needed to better understand the cross-talk between Notch/PTEN/AKT and other pathways in the context of CCN3-induced proliferation and differentiation. Additionally, it is important to investigate whether other signal pathways are involved in the aforementioned mechanisms of proliferation and differentiation.

## 4. Materials and Methods

### 4.1. Animals

The C57BL/6N mouse strain was used for this study and was obtained from the Experimental Animal Center of Xi’an Jiaotong University (Certificate No. 22–9601018). Adult male mice (weight 20–25 g) were used for in vivo experiments, and housing and environmental enrichment were provided in accordance with general standards (12 h/12 h light/dark cycle, food and water ad libitum, and 20–24 °C). The cell culture of hippocampal NSCs was prepared from neonatal mice. All mouse experiments were approved by the Biomedical Ethics Committee of the Health Science Center of Xi’an Jiaotong University (No. XJTUAE2012-1605). All efforts were carried out to minimize animal suffering and to keep the number of used animals to a minimum.

### 4.2. Primary Hippocampal NSCs Culture

The neonatal mouse was anesthetized by isoflurane (5%) and euthanized by 65% CO_2_. The hippocampus tissue was isolated under a dissecting microscope (SZX10, OLYMPUS, Shinjuku, Tokyo, Japan). After washing with ice-cold serum-free DMEM/F12 medium (Gibco, 11320033, Waltham, MA, USA), tissues were disaggregated by P1000 pipettes followed by incubating with ACCUTASE™ (STEMCELL Technologies, 07920, Vancouver, Canada) for 3 min at 37 °C. The tissues were then mechanically dissociated into a single-cell suspension using P1000 pipettes and filtrated through a 40 μm Cell Strainer (Falcon, CORNING, 352340). Subsequently, cells were harvested by centrifugation at 4 °C at 1000 rpm for 3 min (Eppendorf, 5810R, Hamburg, Germany). The cells were seeded at a density of 40,000 cell/mL in non-adhesive T75 flasks with a completed medium (serum-free DMEM/F12 containing 1% N-2 (A13707-01), 2% B-27™ (12587010), 20 ng/mL of epidermal growth factor (EGF, PHG0311) and 10 ng/mL of basic fibroblast growth factor (bFGF, PHG0369)). All these reagents were purchased from Gibco. Cells were then cultured at 37 °C in a CO_2_ incubator (Memmert, INCO108, Schwabach, Germany) with 5% CO_2_/95% air. In this study, 20 ng/mL of EGF and 10 ng/mL of bFGF were replenished every other day. After 3–5 days of culturing, neurospheres within a range of 90–170 μm were observed (P0). The neurospheres were then dissociated into single cells using ACCUTASE™. Single NSCs were cultured at a density of 50,000 cells/mL in a completed medium to obtain secondary neurospheres (P1 cells), which were used for subsequent experiments. Secondary neurospheres were then dissociated into single cells using ACCUTASE™, and single cells were plated on culture plates or coverslips pre-coated with Poly-D-lysine (PDL, 0.1 mg/mL, Gibco, A3890401). The cell proliferation assay was conducted using a completed medium while the cell differentiation assay was conducted using a natural differentiation medium (serum-free DMEM/F12 containing 1% N-2 and 2% B-27™).

### 4.3. Immunostaining

The mice were anesthetized and transcardially perfused with 25 mL of normal saline followed by 50 mL of 4% paraformaldehyde (PFA). Subsequently, the brain was dissected and post-fixed in 4% PFA overnight at 4 °C, followed by paraffin embedding. The hippocampal serial slices were coronally sectioned by a microtome (Leica, RM2235, Wetzlar, Germany) and mounted onto adhesion microscope slides. Following the deparaffinization treatment, the slides were subjected to heat treatment at 95 °C in an antigen retrieval buffer (3 g sodium citrate, 0.4 g citric acid, 1000 mL H_2_O, and pH 6.0) for 30 min and then were washed thrice with 0.01 M PBS. These sections were utilized for immunostaining. For the cultured cell, cells were fixed with 4% PFA for 30 min, followed by washing five times with 0.01 M PBS. Both slices and cell coverslips were permeabilized with 0.3% Triton X-100 (Sigma-Aldrich, X100-100ML, St. Louis, MO, USA) for 15 min, rinsed, and then blocked in 5% bovine serum albumin (Sigma-Aldrich, A8022) with 5% normal goat serum (Sigma-Aldrich, NS02L). For BrdU staining, samples were pretreated with 2N HCl for 30 min at 37 °C before the permeabilization treatment. Thereafter, the samples were neutralized in a 0.1 M borate buffer (pH 8.5) for 15 min. The samples were incubated with primary antibodies (Supplement Table S1) overnight at 4 °C. Following washing with 0.01 M PBS, the samples were incubated with suitable secondary antibodies (Supplement Table S1) for 2 h. The negative controls were just incubated in a blocking buffer instead without primary antibodies. Nuclei were counterstained using a mounting medium containing DAPI or PI. The images were captured using a fluorescent microscope (BX51 + DP71, Olympus, Japan) or a confocal microscope (SP8, Leica, Germany).

### 4.4. Lentiviral Transfection

RNA interference was utilized to reduce *Ccn3* gene expression. The small interfering RNA (siRNA) sequences were designed according to a previous study [44]. The sequences were as follows: siCCN3: 5′-CAAGAGCCCGAGGAAGUAATT-3′; siNC: 5′-CGTACGCGGAATACTTCGATT-3. The lentivirus vector containing siRNA-targeting mouse *Ccn3* (shCCN3) or negative control vectors (shNC) was obtained from Genechem (Shanghai, China). Two lentivirus vectors were used in this study; one expressing eGFP for measuring transfection efficiency, and another that did not express fluorescent proteins for most subsequent experiments.

Cells (5 × 10^4^ cells/well) were seeded onto a 24-well plate and infected with 1 μL of shCCN3 (7 × 10^8^ TU/mL) or 1μL of shNC (1 × 10^8^ TU/mL). One day later, the infected cells were selected with puromycin (1.6 μg/mL) for 24 h.

### 4.5. Stereotaxic Surgery

Adult male mice were anesthetized with pentobarbital sodium (50 mg/kg, IP injection).

The surgical procedures were conducted in sterile conditions, and the body temperature was maintained by using heating pads until the emergence from anesthesia. The mice were placed in a stereotactic frame (69100, RWD Life Science, Shenzhen, China), and a longitudinal incision was made along the midline of the skull to expose the bregma follow by drilling a small hole. Either CCN3 inhibition lentiviral (shCCN3), empty lentivirus (shNC) or CCN3 protein solution was stereotaxically injected in the right DG using the following coordinates: anterior–posterior, -2.0 mm anterior to the bregma; medial–lateral, 1.2 mm; dorsal–ventral, 2.2 mm below the skull surface. For lentiviral injection, a total of 300 nL of the lentiviral solution was injected into the hippocampus at a flow rate of 20 nL/min using a microsyringe pump (KDS Legato™ 130, Holliston, MA, USA). The injector remained in situ for 5 min postinjection. For the protein solution injection, an intracranial cannula (RWD Life Science) was implanted with slight modifications following the method described in our previous study [45]. Briefly, the implanted cannula was secured with three small screws and dental cement. Each mouse was housed alone and allowed to recover for a minimum of a week.

### 4.6. Experimental Treatments

To infuse the protein solution, the mice were anesthetized with 3% isoflurane for induction and 1.5% isoflurane for maintenance by using an inhalant anesthesia machine (RWD Life Science). The intracranial cannula was then connected to a 10 μL microsyringe using a polyethylene tube, and 5 μL of the solution (2 μg/mL) was administered at a flow rate of 0.5 μL/min using a microsyringe pump. The control group was administered the same volume of normal saline. After infusion, the microsyringe remained in situ for an additional 5 min. The internal injection cannula was then slowly pulled out, and the stylet was placed back into the guide cannula. A protein solution or normal saline was injected daily for 10 consecutive days. To demonstrate the dose-dependent effects of CCN3 (R&D System, USA, 1976-NV-050), cells were treated with a series of recombinant mouse CCN3 protein concentrations (5, 10, 20, 50, 100, 200, and 500 ng/mL) for 3 days. FLI-06 (Sigma-Aldrich, 20 Μm, SML0975) or VO-OHpic (Tocris, Bristol, UK, 5 μM, 3591) was used to block the effects of CCN3 or shCCN3 for 3 days, with equivalent DMSO used as vehicle control. All in vitro treatments were performed in at least three independently prepared NSC cultures.

### 4.7. BrdU Incorporation

Cells plated on the PDL-coated coverslips were incubated with 10 μg/mL of BrdU (Sigma-Aldrich, B5002) for 2 h before the end point of the experiment. The BrdU-labeled cells were then visualized using immunostaining and quantified using propidium iodide (PI, Sigma-Aldrich, 537059) staining cells.

### 4.8. Cell Cycle Analysis

Cultured hippocampal NSCs were fixed using pre-cooled 75% ethanol overnight at 4 °C. After washing with 4 °C PBS, cells were stained with 100 μg/mL of PI containing 100 μg/mL of RNase A for 30 min at 37 °C in the dark. Cell cycles were analyzed using FACSCalibur (BD Biosciences, Franklin Lakes, NJ, USA) with excitation at λ488 nm and emission at λ630 nm; 20,000 cells were detected for each sample. The data were collected by FACSort Cellquect software (v7.5.3, BD Biosciences); DNA contents and cell cycle distributions were determined using Modfit LT software (v3.3, BD Biosciences). The proliferation index (PI) was used to evaluate changes to the cell cycle distribution with the following formula: PI = (S + G_2_/M)/(G_0_/G_1_ + S + G_2_/M).

### 4.9. Cell Viability Assay

Cell viability was evaluated by using the Cell Counting Kit-8 (CCK-8; 7Sea, Shanghai, China, C008). Cells (5000 cells/well) were cultured in PDL-coated 96-well plates. On the following day, cells were exposed to a series of CCN3 concentrations (5, 10, 20, 50, 100, 200, and 500 ng/mL), while cells exposed to 0 ng/mL of CCN3 only received solvents.

After incubation for three days, 20 μL of CCK-8 was added to each well and further incubated for two hours. The absorbance at 490 nm was measured using a multimicroplate spectrophotometer (BioTek, Winooski, VT, USA). Three independent experiments were performed, and each experiment consisted of triplicate wells. The data were collected by examining the average of all independent experiments. The results are presented as a percentage of absorbance in the control cells.

### 4.10. TUNEL Staining

Cell apoptosis was detected by using the TUNEL assay kit (Roche Diagnostics, 12156792910, Basel, Switzerland) according to the manufacturer’s instructions. Then, the nuclei were visualized using a mounting medium including DAPI. Microscopic observations and photography were performed in an Olympus BX51 fluorescence microscope, and images were processed using Image-Pro Plus 5.0 software (Olympus).

### 4.11. Western Blot Analysis

Lysis of the cells was carried out using a RIPA lysis buffer that was supplemented with a Protease Inhibitor Cocktail (Roche, 11697498001 Basel, Switzerland) for 15 min on ice. Centrifugation was carried out at 12,000× *g* for 10 min at 4 °C to collect the supernatant. The supernatants were collected, and the protein concentration of the samples was measured using the BCA assay (Pierce, 23227, Waltham, MA, USA,). The electrophoresis of each protein (20–40 μg) was conducted using 10–12% SDS-PAGE and polyvinylidene fluoride (PVDF) membranes (BioRad, 1620177, Hercules, CA, USA). The membranes were blocked in 5% non-fat milk dissolved in a Tris-HCl buffer that contained 0.05% Tween-20 (TBST) for 2 h at room temperature. Subsequently, the membranes were probed with specific primary antibodies overnight at 4 °C (Supplement Table S1). Membranes were then incubated in HRP-conjugated secondary antibodies (Supplement Table S1) for 2 h at room temperature. After washing with TBST, immunoreactive bands were visualized with an enhanced chemiluminescent substrate according to the manufacturer’s protocol (Pierce, 34076). The bands were collected using GeneGnomeXRQ (Syngene, Bengaluru, India) and analyzed using ImageJ 3.5 software. The expression levels of target proteins were analyzed and then normalized relative to the housekeeping β-Actin. All Western blot data were presented in samples from at least 3 independent experiments.

### 4.12. Statistical Analysis

Statistical analyses were performed using GraphPad Prism 5.0 software. The data were presented as mean ± standard deviation. Normality and homoscedasticity were checked before a comparison of data. One-way ANOVA was used for significance testing. Comparisons between the two groups were conducted by Fisher’s PLSD test. The Kolmogorov–Smirnov test was used for normality and homogeneity. The data were shown as mean  ±  standard deviation, and *p* < 0.05 was considered as a statistically significant difference.

## 5. Conclusions

Our study demonstrates that the supplement of CCN3 promotes the proliferation of mouse hippocampal NSCs and suppresses their differentiation into neurons. CCN3 was found to upregulate NICD expression and downregulate PTEN expression, which in turn promotes the activation of AKT. In contrast, *Ccn3* knockdown reduces NSC proliferation, stimulates neuronal differentiation, and inhibits Notch/PTEN/AKT activation. Additionally, Notch and PTEN inhibitors counteract changes in NSC behavior resulting from CCN3 protein expression. Our findings highlight the potential of CCN3 as a therapeutic target for repairing and regenerating the CNS, particularly in the context of hippocampal dysfunction.

## Data Availability

The data used and/or analyzed during the current study are available from the corresponding author upon reasonable request.

## Supplementary Materials

The following supporting information can be downloaded at https://www.mdpi.com/article/10.3390/ijms241210324/s1.

## Abbreviations

NSCs	neural stem cells
SGZ	subgranular zone
CCN	cellular communication network
CCN3	cellular communication network protein 3
DG	dentate gyrus
PI	proliferation index
NOV	nephroblastoma overexpressed
Tuj1	class III beta tubulin
DCX	Doublecortin
BrdU	5-Bromo-2′-deoxyuridine
CCK-8	Cell Counting Kit-8
MSCs	mesenchymal stem cells
HSC	Hematopoietic stem cell
GNP	granule neuron precursors
EGF	epidermal growth factor
bFGF	basic fibroblast growth factor
PDL	Poly-D-lysine
PFA	paraformaldehyde
CNS	central nerve system
LRP1	low-density-lipoprotein receptor-related protein 1
PIP3	Phosphatidylinositol-3,4,5- trisphosphate
PIP2	Phosphatidylinositol (4,5)- bisphosphate
PLD1	Phospholipase D1
CTGF	connective-tissue growth factor
WISP1	Wnt1 inducible signaling pathway protein 1
