# Supplementary material for: CCN3/NOV Regulates Proliferation and Neuronal Differentiation in Mouse Hippocampal Neural Stem Cells via the Activation of the Notch/PTEN/AKT Pathway
Source: Int J Mol Sci. 2023 Jun 19;24(12):10324. doi: 10.3390/ijms241210324 (PMC10299577; doi:10.3390/ijms241210324)
Supplement: Supplementary file 1 [file ijms-24-10324-s001.zip › ijms-2432346-supplementary.pdf]

Table S1. Antibodies used during the study.

Antigen	Source and host species	Concentration	Catalog No.
anti-SOX2	Abcam, rabbit polyclonal antibody	1:200	ab97959
anti-nestin	Milliproe, mouse monoclonal antibody	1:200	MAB5326
anti-Tuj1	Milliproe, mouse monoclonal antibody	1:200 (IF)<br>1:500 (WB)	MAB1637
anti-GFAP	Abcam, rabbit polyclonal antibody	1:1000	ab7260
anti-NG2	Milliproe, rabbit monoclonal antibody	1:200	AB5320
anti-CCN3	Abcam, rabbit polyclonal antibody	1:200 (IF)<br>1:1000 (WB)	ab137677
anti-DCX antibody	Abcam, rabbit polyclonal antibody	1:200	ab18723
anti-Ki67	Invitrogen, rat monoclonal antibody	1:1000	14-5698-80
anti-BrdU	Abcam, sheep polyclonal antibody	1:200	ab1893
anti-phospho-AKT	Cell Signaling Technology, rabbit monoclonal antibody	1:1000	4060
anti-Cleaved Notch1	Cell Signaling Technology, rabbit monoclonal antibody	1:1000	4147
anti-HES1	Cell Signaling Technology, rabbit monoclonal antibody	1:1000	4147
anti-AKT	Cell Signaling Technology, rabbit polyclonal antibody	1:1000	11988
anti-PTEN	Cell Signaling Technology, rabbit polyclonal antibody	1:1000	9552

anti-β-actin	Sigma-Aldrich, mouse monoclonal antibody	1:5000	A1978
anti-mouse IgG (H+L) secondary antibody, Alexa Fluor 488	Invitrogen, goat polyclonal antibody	1:500	A-11001
anti-rabbit IgG (H+L) secondary antibody, Alexa Fluor 594	Invitrogen, goat polyclonal antibody	1:500	A-11012
anti-Rat IgG (H+L) Highly Cross-Adsorbed secondary antibody, Alexa Fluor 594	Invitrogen, donkey polyclonal antibody	1:500	A-21209
anti-Rabbit IgG (H+L) secondary antibody, Alexa Fluor 488	Invitrogen, donkey polyclonal antibody	1:500	R37118
anti-sheep IgG (H+L) secondary antibody, Alexa Fluor 594	Invitrogen, donkey polyclonal antibody	1:500	A-11016
HRP-conjugated anti-Mouse IgG	Sigma-Aldrich, goat polyclonal antibody	1:10000	AP130P
HRP-conjugated anti-rabbit IgG	Sigma-Aldrich, goat polyclonal antibody	1:10000	AP307P
